# Supplementary material for: Sustained activation of the Aryl hydrocarbon Receptor transcription factor promotes resistance to BRAF-inhibitors in melanoma
Source: Nat Commun. 2018 Nov 14;9:4775. doi: 10.1038/s41467-018-06951-2 (PMC6235830; doi:10.1038/s41467-018-06951-2)
Supplement: Supplementary file 1 — Supplementary Information [file 41467_2018_6951_MOESM1_ESM.pdf]

Supplementary Figure 1

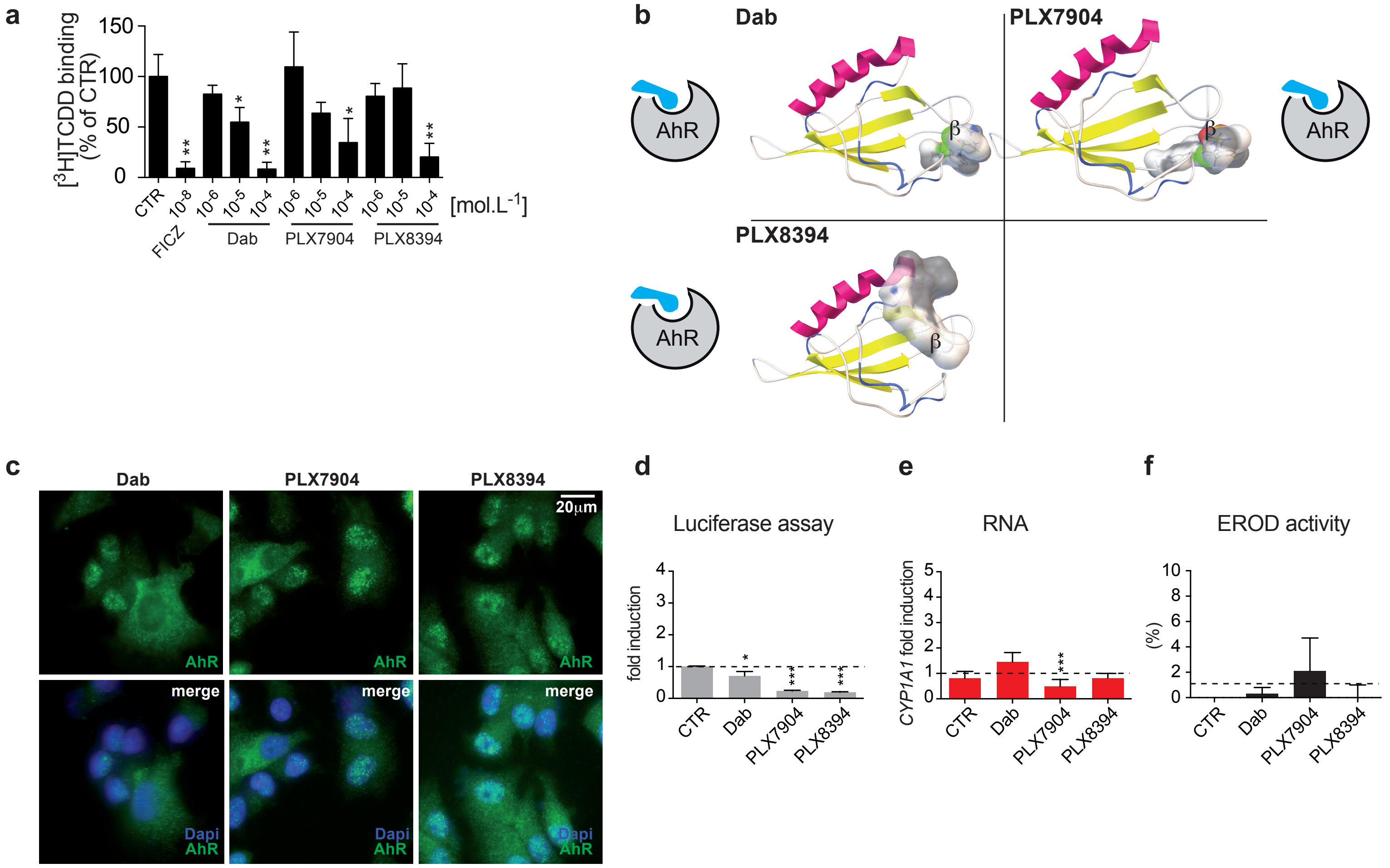

Supplementary Figure 1: BRAF-V600E inhibitors bind to AhR and antagonizes canonical AhR pathway.

a, Competitive binding of FICZ, dabrafenib (Dab), PLX7904 or PLX8394 to AhR. Hepatic cytosol containing AhR was incubated with [^{3}H]TCDD in the presence of DMSO (1%), or FICZ or increasing concentrations of BRAFi (10^{-6} to 10^{-4} mol/L^{-1}).

b, Proposed binding mode of Dab, PLX7904 and PLX8394 into the homology model of PAS-B of AhR. Free binding energy is reported in Table 1.

c, AhR nuclear translocation in response to Dab (100 nM), PLX7904 (1 μM) or PLX8394 (1 μM) in MCF7 cells 1 h after treatment. AhR in green (IHC) and nucleus staining in blue.

d-f, BRAFi do not activate canonical AhR transcriptional activity. **d**, Evaluation of AhR transcriptional activity related to AhR/ARNT binding sites (XRE) using p3XRE-luciferase constructs. MCF7 cells were exposed to Dab (100 nM), PLX7904 (1 μM) or PLX8394 (1 μM) or vehicle (DMSO) for 6 h.

e, BRAFi do not induce *CYP1A1* mRNA. MCF7 cells were either untreated or treated with Dab (100 nM), PLX7904 (1 μM) or PLX8394 (1 μM) for 15 h.

f, BRAFi do not induce EROD activity. EROD enzymatic activity is associated with members of the cytochrome P450 1 family. MCF7 cells were either untreated or treated with Dab (100 nM), PLX7904 (10 μM) or PLX8394 (10 μM) or vehicle (DMSO) for 6 h.

For the different experiments, data are expressed in arbitrary units, comparatively with the value found in DMSO-treated cells, arbitrarily set to 1 and correspond to the means +/- s.d. of three independent experiments. Statistical analysis was performed using unpaired t-test (PRISM6.0®) *, $p<0.05$; **, $p<0.01$; ***, $p<0.001$.

Supplementary Figure 2

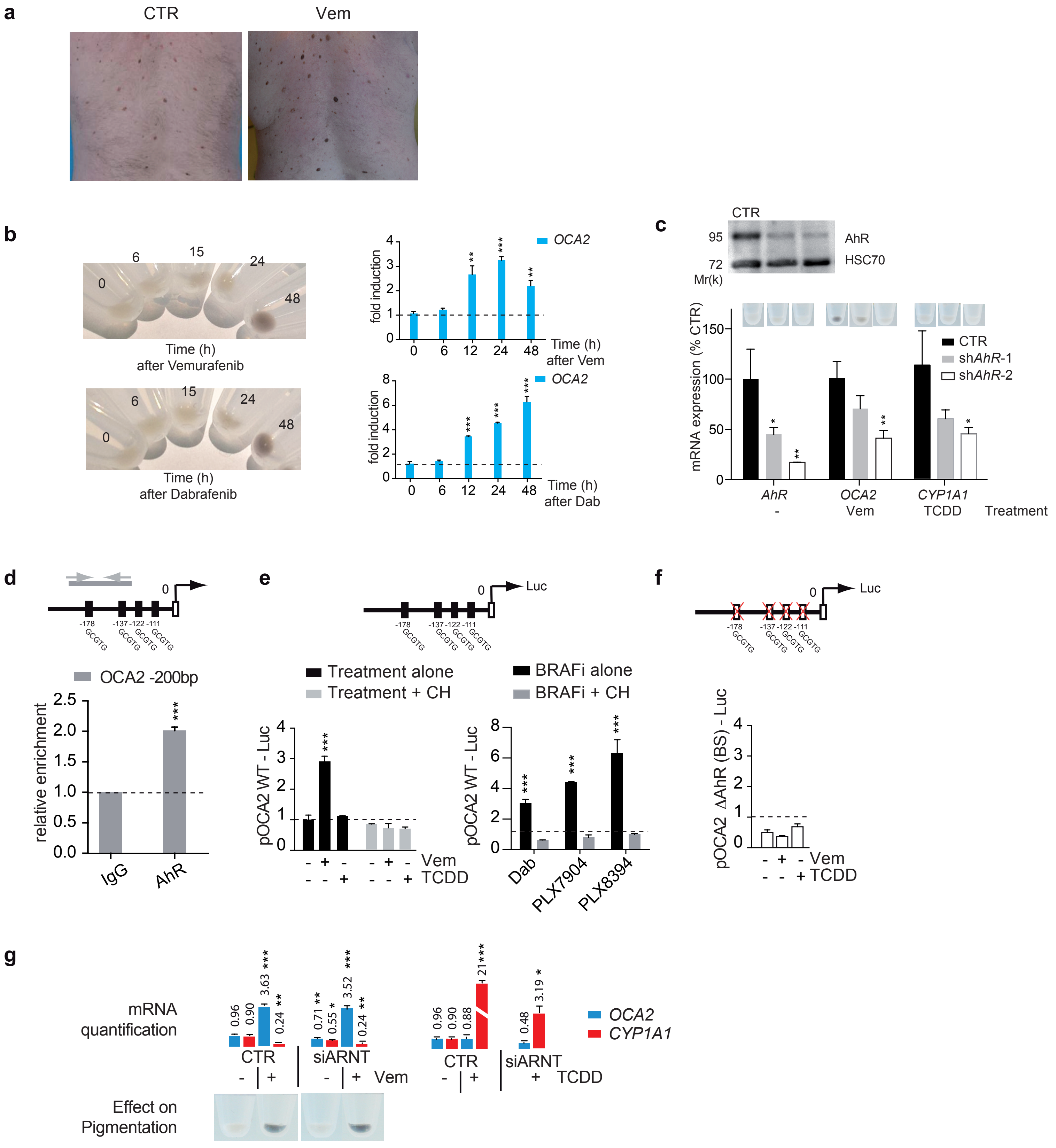

Supplementary Figure 2: AhR-OCA2 axis is associated to pigmentation.

a, Vem induces pigmentation *in vivo* Picture of nevi from patient treated with Vem.

b, Kinetic of 501Mel cells pigmentation and *OCA2* mRNA induction in response to Vemurafenib (1 μM) or Dabrafenib (100 nM) exposure (in hour).

c, *AhR* knock down reduces *OCA2* mRNA and *CYP1A1* mRNA expression levels in 501Mel melanoma knock-downed for AhR by two shRNA in response respectively to Vem **(**1 μM) and TCDD (10 nM) (48 h).

d-f, Vem-activated AhR induces pigmentation by transactivating *OCA2* promoter *via* AhR binding sites. **e,** For ChiP experiment, histograms represent the mean +/- s.d. relative occupancy of AhR onto *OCA2* promoter referred to non-specific IgG antibody for three independent experiments in 501Mel cell line. Promoter activity has been evaluated using pOCA2-Luc wild-type **(e)** or mutated AhR Binding sites constructs (ΔAhR BS) **(f)** after Vem (1 μM) or TCDD (10 nM), or vehicle (DMSO) for 6 h in the presence or absence of the specific AhR antagonist CH-223191 (10 μM).

g, Pigmentation and *OCA2* induction in response to Vem depend of AhR expression level but not to ARNT. Data are representative of knock-down 501Mel cells for ARNT using siRNA targeting ARNT. Control or depleted 501Mel cells for ARNT were treated (+) with Vem (1 μM) or TCDD (10 nM) or vehicle (-). *OCA2* and *CYP1A1* mRNA levels have been analysed using RT-qPCR. Data are expressed in arbitrary units, comparatively with the value of expression level found in vehicle-treated cells (CTR). Western blot or RT-qPCR analyses have been performed to confirm AhR depletion in cells for ARNT (data not shown)**.**

For the different experiments, data are expressed in arbitrary units, comparatively with the value found in DMSO-treated cells (CTR), arbitrarily set to 1 and correspond to the means +/- s.d. of three independent experiments. Statistical analysis was performed using unpaired t-test (PRISM6.0®) *, $p<0.05$; **, $p<0.01$; ***, $p<0.001$.

Supplementary Figure 3

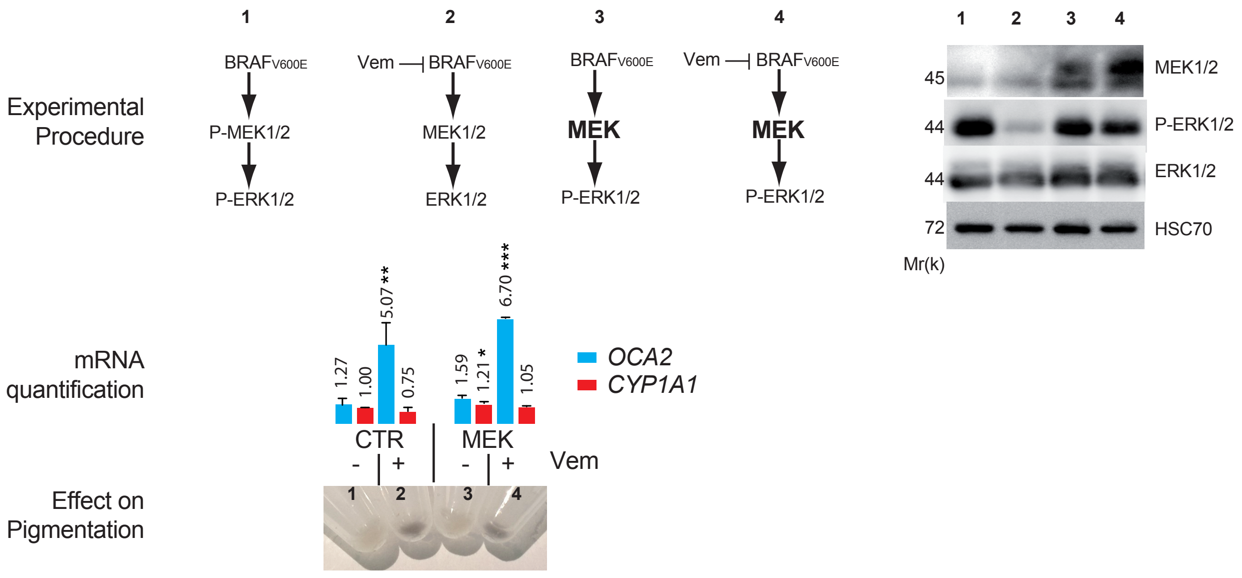

Supplementary Figure 3: AhR reprograming by Vem is ERK-independent.

501Mel cells were or not transfected with dominant form of MEK kinase to prevent the effect of Vemurafenib on inhibition of MER/ERK pathway. 24 h after transfection, 501Mel cells were untreated or treated with 1 μM Vemurafenib (Vem) for 48 h. *OCA2* and *CYP1A1* mRNA levels were analyzed using RT-qPCR (middle). Activation or inhibition of MEK/ERK pathway was confirmed by Western blot analysis of MEK1/2, P-ERK1/2, ERK1/2, and HSC70 (as loading control) in protein extracts from treated 501Mel cells.

Supplementary Figure 4

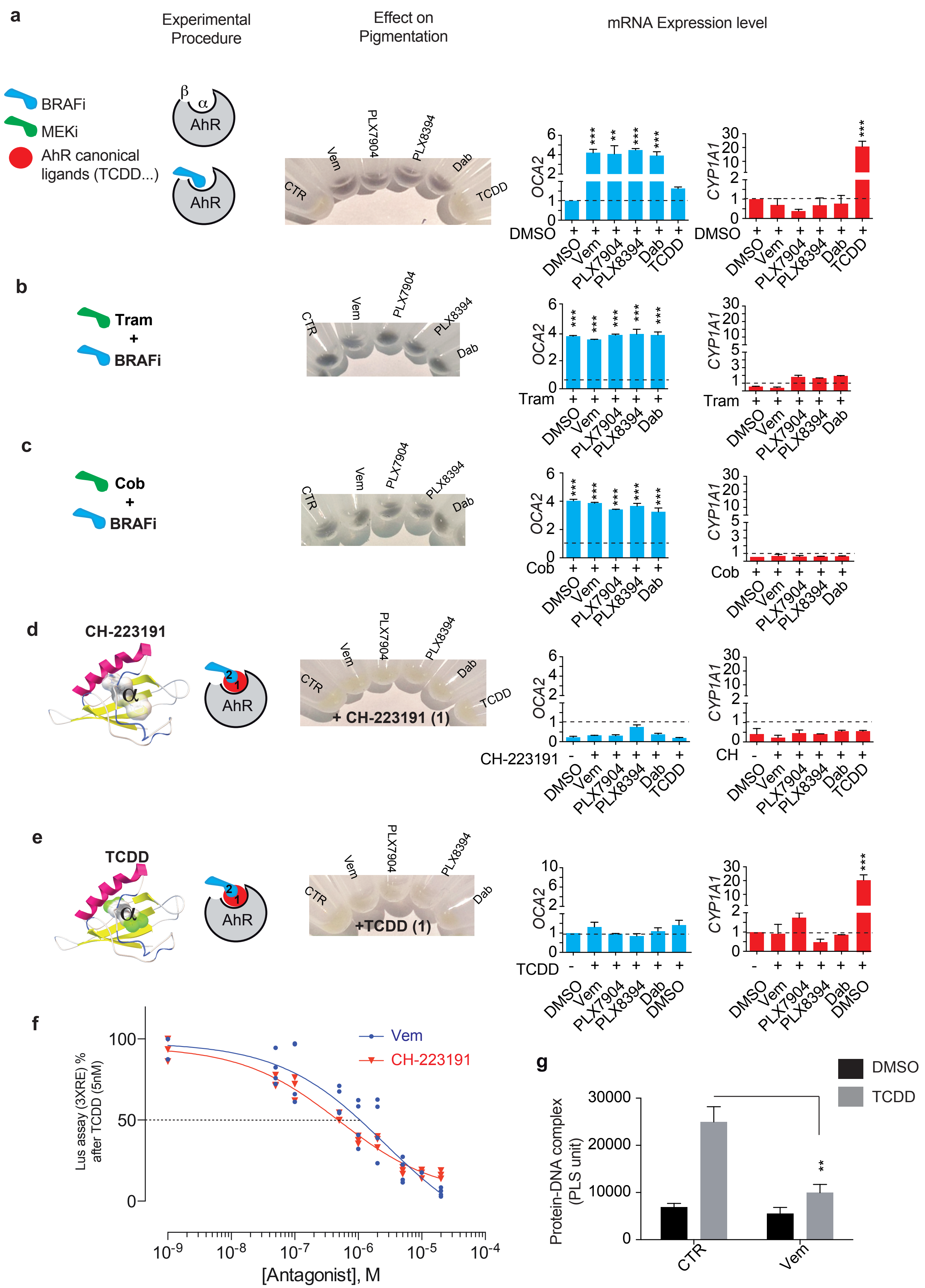

Supplementary Figure 4: AhR reprogramming by BRAFi is druggable by targeting the α-pocket of AhR.

Pigmentation and *OCA2* inductions in cells exposed for 48 h to BRAFi Vemurafenib (Vem) (1 μM), PLX7904 (1 μM), PLX8394 (1 μM), Dabrafenib (Dab) (100 nM) alone **(a)** or in competition with MEK inhibitors Trametinib (1 μM) **(b)**, Cobimetinib (1 μM) **(c)**, AhR ligands CH-223191 (10 μM) **(d)** or TCDD (10 nM) **(e)**. BRAFi and/or MEKi alone induced *OCA2* expression and pigmentation in contrast to combination with CH-223191 or TCDD. *CYP1A1* was only induced by TCDD, a potent AhR agonist (α-pocket). Pretreatment (2 h) with AhR antagonist CH-223191 (α-pocket) prevented both BRAFi and TCDD effects **(d)**. Similar results have been obtained when 501Mel cells have been pre-treated with TCDD instead of AhR antagonist **(e)**. It is important to note that *CYP1A1* was not induced in this condition by TCDD (TCDD followed by BRAFi exposure). Vem could be considered as AhR antagonist considering competition assay with TCDD using p3XRE-luciferase assays.

(f) 501Mel cells have been transfected with p3XRE-luciferase construct and induced simultaneously with TCDD (5 nM) and increasing doses of Vem or CH-223191 to compete with AhR agonist.

(g) Vem prevents AhR binding on XRE motifs onto DNA induced by TCDD. *In vitro* synthesized wild-type mAhR and ARNT were incubated in the presence of solvent control (DMSO, 1%, vol/vol) or TCDD (20 nM) in the presence or not of Vemurafenib (1 μM) for 2 to 2.5h and analysed by gel retardation assay. Gels were visualized and specific band were quantified (n=3).

For the different experiments, data are expressed in arbitrary units, comparatively with the value found in DMSO-treated cells (CTR), arbitrarily set to 1 and correspond to the means +/- s.d. of three independent experiments. Statistical analysis was performed using unpaired t-test (PRISM6.0®) *, $p<0.05$; **, $p<0.01$; ***, $p<0.001$.

Supplementary Figure 5

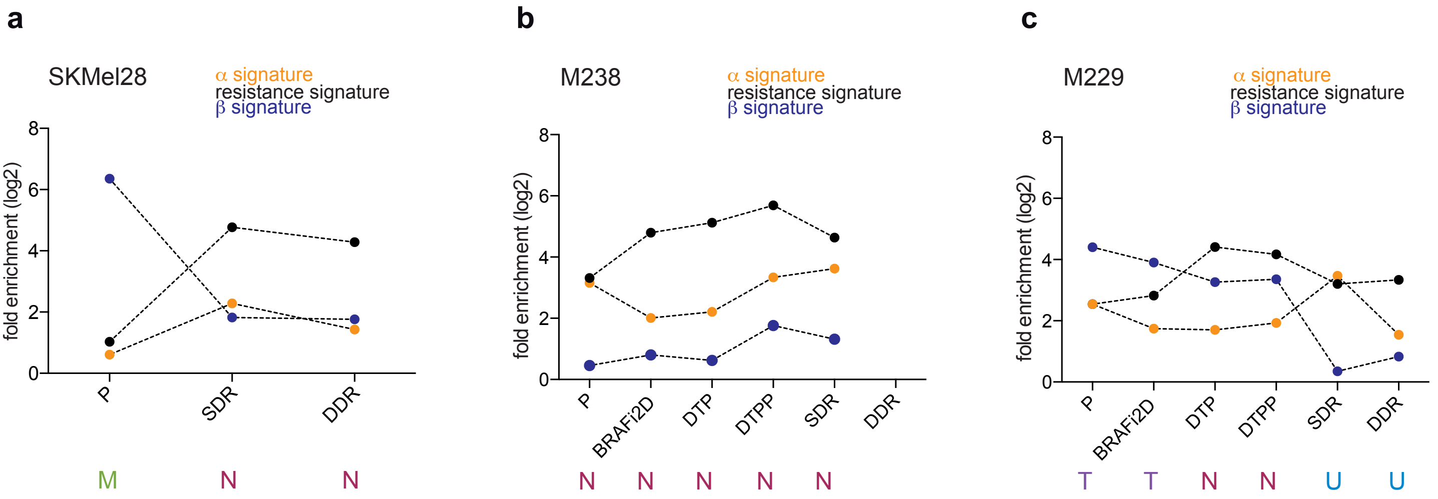

Supplementary Figure 5: AhR-signature correlates with resistance acquiring during after long-term treatment with single-drug (i.e. BRAFi) or double-drug (i.e. BRAFi+MEKi)

a-c, Fold expression level (log2) for average β-, α–, resistance and differentiated state signatures in parental (P) cell lines (SKMel28, M234 and M229) treated with DMSO/vehicle, temporal sub-populations (2 d, DTP, DTPP) (days to weeks on BRAFi : PLX4032), and long-term sub-lines (months to years on BRAFi or BRAFi+MEKi : PLX4032+Selumitinib) resulting in single-drug resistant (SDR) or double-drug resistant (DDR) lines. (GEO, GSE75299 [1]). To derive DTPP clones, parental melanoma cells seeded at low density were treated with drugs as described [1] every 2-3 days for 3-6 weeks, SDR and DDR sub-lines were derived as described [2–4].

Supplementary Figure 6

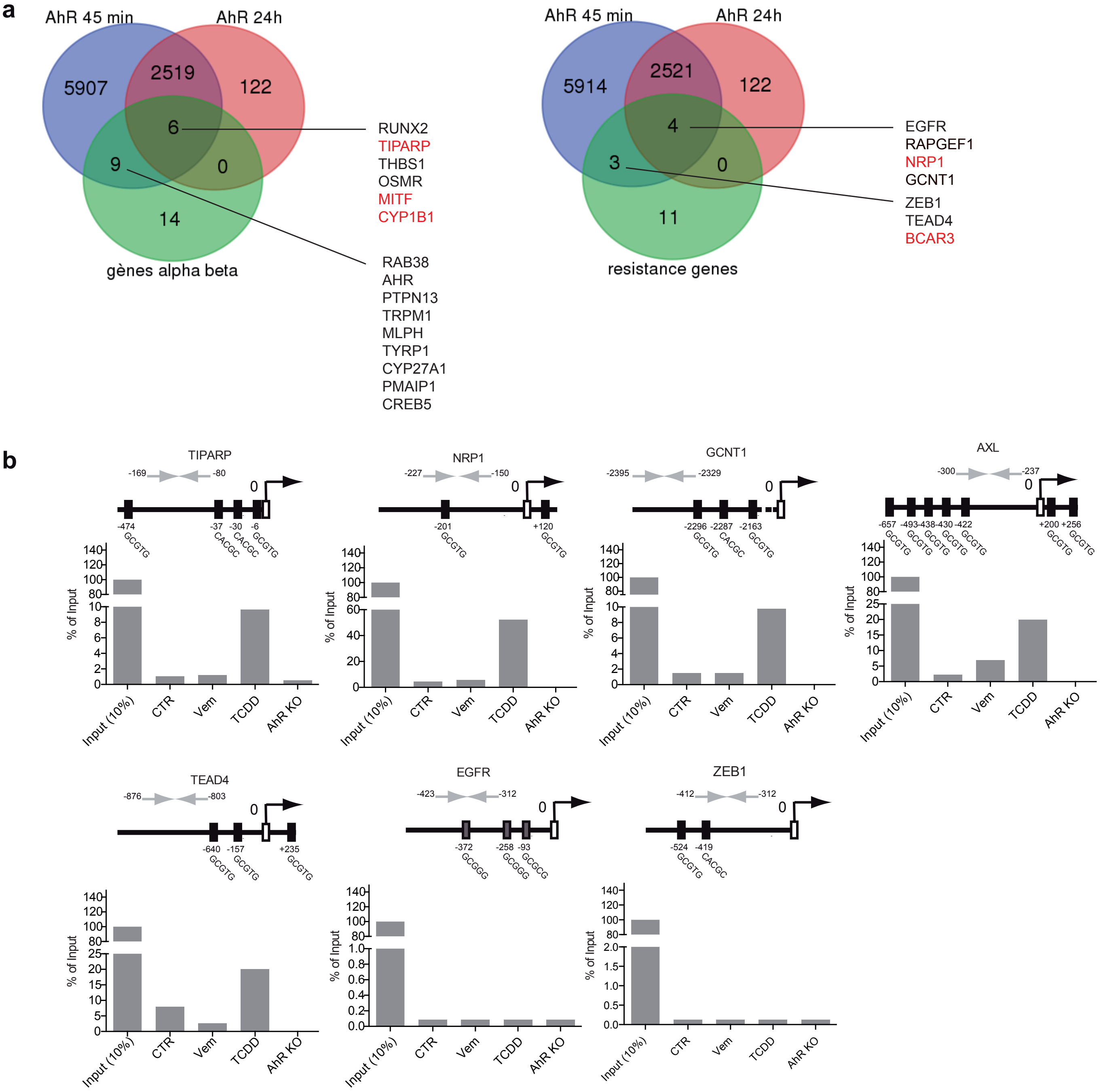

Supplementary Figure 6: AhR binds to resistance genes promoter in response to α-ligand

a, Venn diagram representing the overlapping between α- and β- or resistance genes with dataset for ChIPseq experiments performed by Mathews'lab for AhR transcription factor 45 min to 24h after induction by TCDD (10 nM) of MCF7 cells (GSE90550 [5]).

b, ChiP experiment has been performed using specific AhR antibody in 501Mel cells and in knocked-down cells (as negative control) treated for 1h with TCDD (10nM) or 6h with Vem (1 μM) (n=1). Values represent the percentage of enrichment of the transcription factor onto proximal promoter of target genes, containing XRE motifs, compared to Input.

Supplementary Figure 7

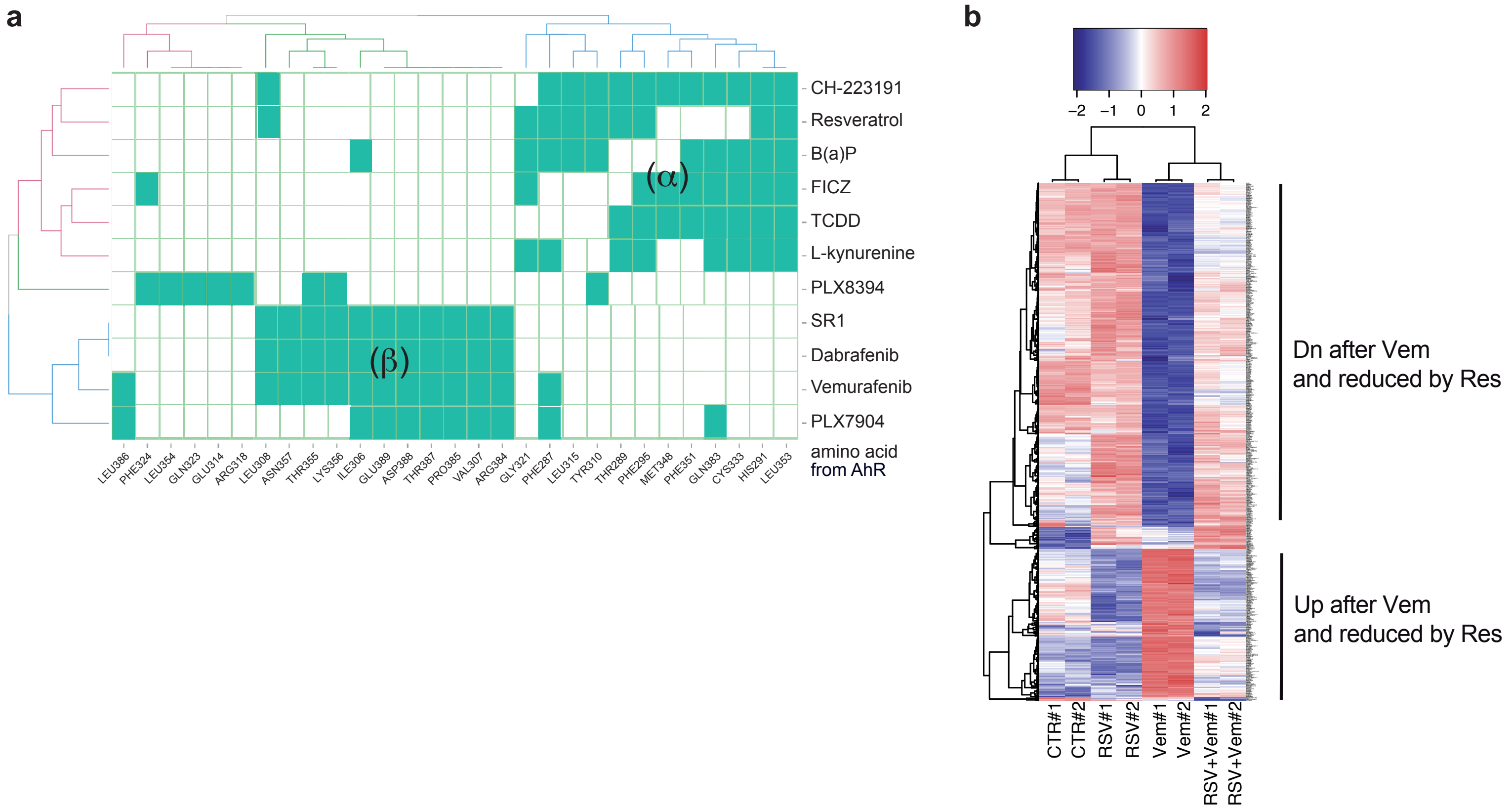

Supplementary Figure 7: The AhR antagonist Resveratrol blocks Vem induced AhR reprograming (β-signature).

a, Heat map representing hierarchical clustering of AhR ligands and their putative interactions with amino-acids of PAS B domain of AhR. BRAFi clustered together.

b, Gene expression profile of the 501Mel cells exposed to vehicle, Vem (1 μM), RSV (5 μM) or RSV (5 μM) + Vem (1 μM) (n=2) for 48 h. Heatmap focused on differentially expressed genes in function of treatment.

Supplementary Figure 8

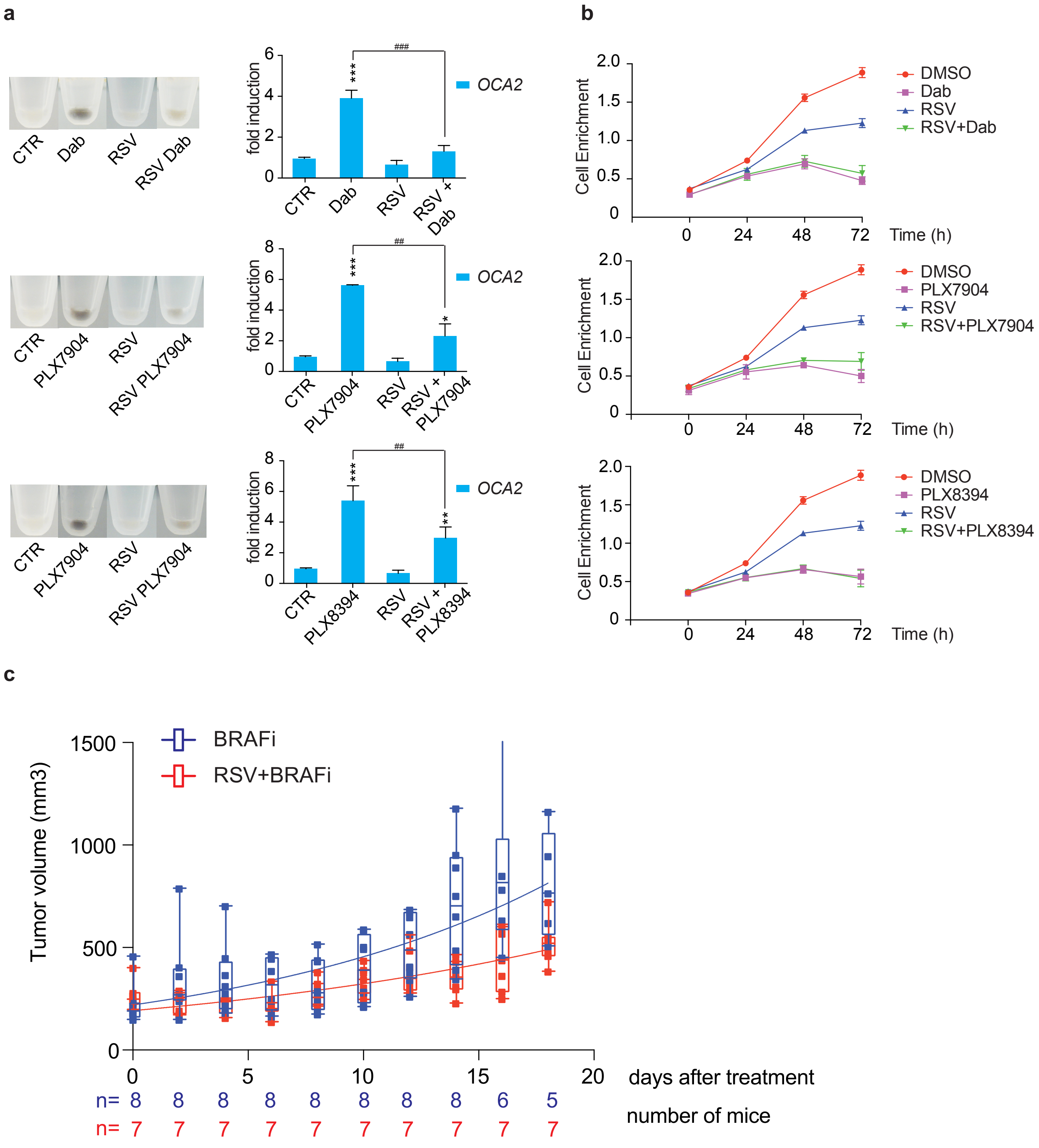

Supplementary Figure 8: Resveratrol prevents AhR reprogramming and maintains antiproliferative effects of BRAFi.

a, Pigmentation analyses and *OCA2* mRNA expression levels (n=3) in 501Mel cells exposed 48 h to BRAFi Dabrafenib (Dab) (100 nM), PLX7904 (1 μM), PLX8394 (1 μM) alone or pretreated for 2 h with RSV (5 μM).

b, RSV preserved antiproliferative effect of BRAFi in 501Mel cells. After different days after treatment, 501Mel cell density was evaluated by methylene blue staining followed by quantification at 620 nm (n=2).

c, PDX tumor volumes for individual mice after daily treatment with Dabrafenib (30 mg/Kg) (n=8) or in combination with RSV (40 mg/Kg) (n=7).

Supplementary Tables

Supplementary Table 1 : Binding Energy of different ligands for PAS-B domain of AhR and AA close contact evaluated by docking experiments.

Chemicals		Binding Energy	H-Bond	Pi-Cation interaction	Close Contact
**TCDD**	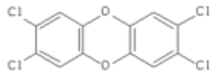	**-7.68**			THR289<br>HIS291<br>PHE295<br>CYS333<br>HIS337<br>MET348<br>PHE351<br>LEU353<br>GLN383
**FICZ**	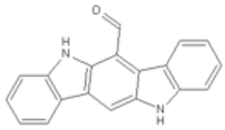	**-9.38**	HIS291<br>GLN383		HIS291<br>PHE295<br>GLY321<br>PHE324<br>CYS333<br>SER336<br>HIS337<br>MET348<br>PHE351<br>LEU353<br>GLN383
**BaP**	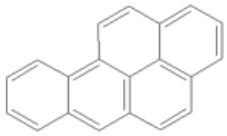	**-9.22**			PHE287<br>HIS291<br>LEU306<br>TYR310<br>LEU315<br>GLY321<br>CYS333<br>PHE351<br>LEU353<br>VAL363<br>GLN383
**CH-223191**	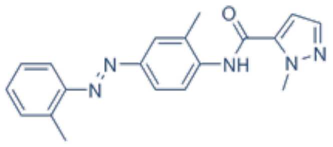	**-8.05**			PHE287<br>THR289<br>HIS291<br>PHE295<br>LEU308<br>TYR310<br>LEU315<br>TYR332<br>CYS333<br>SER336<br>HIS337<br>MET348<br>PHE351<br>LEU353<br>GLN383

Chemicals		Binding Energy	H-Bond	Pi-Cation interaction	Close Contact
**Vemurafenib**	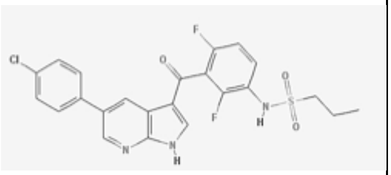	**-8.01**	ILE306 THR387 LEU386		PHE287<br>ILE306<br>VAL307<br>LEU308<br>THR355<br>LYS356<br>ASN357<br>ARG384<br>PRO385<br>LEU386<br>THR387<br>ASP388<br>GLU389
**Dabrafenib**	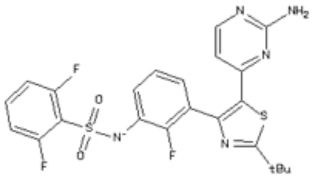	**-8.07**	ILE306 THR387		ILE306<br>VAL307<br>LEU308<br>THR355<br>LYS356<br>ASN357<br>ARG384<br>PRO385<br>THR387<br>ASP388<br>GLU389
**PLX7904**	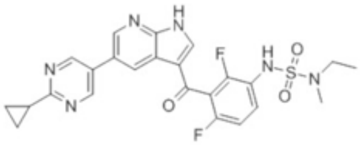	**-6.79**	ILE306 THR387 LEU386		PHE287<br>ILE306<br>VAL307<br>GLY309<br>GLN383<br>ARG384<br>PRO385<br>LEU386<br>THR387<br>ASP388<br>GLU389
**PLX8394**	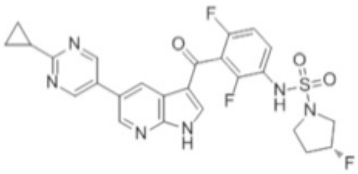	**-7.96**	GLN323 THR355 His326 ILE325	ARG316	TYR310<br>GLU314<br>ARG318<br>GLN323<br>PHE324<br>ILE325<br>HIS326<br>ALA327<br>MET330<br>LEU354<br>THR355<br>LYS356<br>TRP360
**Resveratrol**	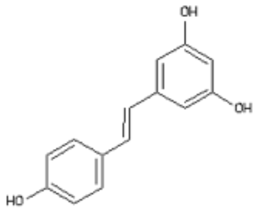	**-6.39**	CYS300 GLY321		PHE287<br>THR289<br>HIS291<br>PHE295<br>CYS300<br>LEU308<br>TYR310<br>LEU315<br>GLY321<br>ILE325<br>LEU353

common AA inside the pocket

common AA outside the pocket

common AA between AhR ligand and BRAFi

Supplementary Table 2 : siRNA, shRNA and CRISPR Cas9 sequences.

IDT DNA siRNA		
siCTR-siNC1	Sens 5'- 3'	CGUUAAUCGCGUAUACGCGUA
mOCA2 mm.Ri.OCA2.13.1	Sens 5'- 3'	GCCCUACUAAUAAAGAUGCUGGAACCAUCUUUAUUA
hARNT hs.Ri.ARNT.13.1	Sens 5'- 3'	UACGCAUGGCAGUUUCUCACAUGAA
hARNT hs.Ri.ARNT.13.3	Sens 5'- 3'	GACCUGAAAUUGUAUAGUGUUGATT

Origene shRNA human AhR		
shAhR #1	Sens 5'- 3'	CACTAGTGGAAAAGACTCTGCTACCACATCCA
shAhR #2	Sens 5'- 3'	CAGCAACAGTCCTTGGCTCTGAACTCAAG
shAhR #3	Sens 5'- 3'	GTAATCAGCCTGTATTACCACAACATTCCA

Sigma shRNA murin AhR		
shCTR	Sens 5'- 3'	SHC002V
shAhR TCRN0000055410	Sens 5'- 3'	CCGGGCTGGATAATTCATCTGGTTTCTCGAGAAACCAGATGAATTATCCAGCTTTTTG
shAhR TCRN0000218025	Sens 5'- 3'	GTACCGGGTCAAGCCTGTTAGCTATATTCTCGAGAATATAGCTAACAGGCTTGACTTTTTTG

CRISPR CAS9 sg sequence		
hAhR #1	Sens 5'- 3'	GGATAACTGTAGAGCAGCAA
hAhR #2	Sens 5'- 3'	CCCCTACTGAAAGAAACGGA

Supplementary Table 3 : Primer sequences for RT-qPCR and for ChIP-qPCR.

	RT-qPCR primers				
	Genes				
Pigmentation signature	TYR	Forward	cctctagtcctcacaaggtctgca	Reverse	gtgggatacgagccaattcga
	MLANA	Forward	gagaaaaactgtgaacctgtggt	Reverse	aaggtggtggtgactgttctg
	MITF	Forward	atgctggaaatgctagaatataatcactatc	Reverse	gggcttgctgtatgtggtacatg
	SLC45A2	Forward	tccactaccatgccctcttc	Reverse	cccagtctatagcacccaaaa
	TRPM1	Forward	ggacatctttggtgtcaacaagt	Reverse	ccacaaagtacagcatgtcgat
	MLPH	Forward	cacagttgtgcctcccttg	Reverse	ggcctcctcctctacatcg
	SNCA	Forward	gagtggccattcgacgac	Reverse	ccctgtttggttttctcagc
	Rab27a	Forward	gctgccaatgggacaaacata	Reverse	caccgttccattcgcttcat
	GPR143	Forward	cactgatgccccatgaaaa	Reverse	tgtgctggcatcagaacc
	TYRP1	Forward	ccagagggtctcatcatagtcaggag	Reverse	atatccagggcccggaca
	MC1R	Forward	gctcaaggaggtgctgacat	Reverse	cttcacatcccagctgacg
	OCA2	Forward	ggagtttgaacttgaccagga	Reverse	agtttccgcagcctgaagt
AhR signature	AhR	Forward	accagcctcaggatgtgaac	Reverse	tcattatgctgtacaagtcactgttt
	CDK2	Forward	gggctcgaaatattattccaca	Reverse	cagaatctccagggaacagg
	AhRR	Forward	agagggcaccttctgcaaac	Reverse	ctcagctctcgcttgatctt
	TIPARP	Forward	tctcaggctcccgttcag	Reverse	tggtttccatttccataatgtg
	INHBA	Forward	agctcagacagctcttaccaca	Reverse	ttttccttctcctcttcagca
	PMAIP1	Forward	uagcgauaaacacaucaa	Reverse	cuauccagcaaagcucuaa
	THBS1	Forward	caatgccacagttcctgatg	Reverse	tggagaccagccatcgtc
	REEP2	Forward	acgctcacggatatagtgctc	Reverse	gctggagcccttggtgta
	CYP1B1	Forward	acgtaccggccactatcact	Reverse	ctcgagtctgcacatcagga
	OSMR	Forward	tgagtttttcatcactccattca	Reverse	gatatgaatcagcatcgaggagt
	CYP1A1	Forward	accttccctgatccttgtga	Reverse	gatcttggaggtggctgct
Resistant genes	BIRC3	Forward	gatgaaaatgcagagtcatcaatta	Reverse	catgattgcatcttctgaatgg
	GCNT1	Forward	cctgacagcatgtgaagtgc	Reverse	cttgaaatgaagagcagcaca
	LPAR5	Forward	aaagtgtggaagccagggta	Reverse	cactgccaccccttaaagaa
	EGFR	Forward	ttcctcccagtgcctgaa	Reverse	ggttcagaggctgattgtgat
	ZEB1	Forward	ttagacacaagcgagaggatca	Reverse	tgaatctgaatttgtttctaccaca
	NRP1	Forward	aataaccacatttcacaagaagattg	Reverse	tcatcaattttaatttctgggttct
	LPAR1	Forward	ccatctctacttccatccctgtaa	Reverse	actcgttgtagaagcactgtgg
	ITGB3	Forward	cgctaaatttgaggaagaacg	Reverse	gaaggtagacgtggcctcttt
	AXL	Forward	cgtaacctccacctggtctc	Reverse	tcccatcgtctgacagca
	NGFR	Forward	tcatccctgtctattgctcca	Reverse	tgttctgcttgcagctgttc
	ITGA9	Forward	ttgttggtgggaatcctcat	Reverse	accttcggcgaaagaagc
	SNX13	Forward	tgttaaaagatcctagtttcagaggat	Reverse	gcttcctgtaggagacccatt
	PCDH7	Forward	ctaccaccagccaacacattt	Reverse	tggaattcagccaaacacag
	BCAR3	Forward	agaaaggaggaagaggcaaga	Reverse	tctgggaaggcttgcaaat
	BRI3	Forward	accgagatcctgctgacg	Reverse	cccctcttggatcgatgtt
	TEAD4	Forward	gctccggacagggaagac	Reverse	caaagctccttgccaaaact
	WDR24	Forward	aaggacatcgacgagcagcac	Reverse	gttggacacgttccagagg
	MAP3K11	Forward	tgggatgcactacctgcac	Reverse	tgctccatgtcgtcactctc
	HMGXB3	Forward	aggggacaagtacctccagtc	Reverse	ggggtaagcaggtcaactcc
	rRNA 18S	Forward	ccggggaggtagtgacga	Reverse	aaggatttaaagtggactcattcca
	MARK3	Forward	aaaagtaaaattggcaaagacatatcc	Reverse	ttttgtcaattatttttattgcaacc

ChIP-qPCR primers				
Genes				
pTIPARP	Forward	tggggagtaggcaaataaaca	Reverse	ttccgagaagacccagacac
pNRP1	Forward	aacttggcgactgggaatc	Reverse	cgcactcgccactctctt
pGCNT1	Forward	ctggagtgcagtggtgtgat	Reverse	ggagggatcacttgaacctg
pAXL	Forward	cctgagctgagaggggagt	Reverse	gtccctctgggctctgtgt
pTEAD4	Forward	aagcgtctccaaacaaggag	Reverse	acacatgcccaggagtctct
pEGFR	Forward	accctggcacagatttgg	Reverse	tgaggagttaatttccgagagg
pZEB1	Forward	ggcgtgggactgatggta	Reverse	attctccctgtaccctgtgc
pOCA2	Forward	gcaaggctctctgttttcca	Reverse	ccccctcttgagaatggtc

Supplementary Table 4 : Statistic data sources.

Figure 1

Figure 1a				
**%**	**Mean**	**SD**	**n**	**P value**
**CTR**	100	21,821	3	
**FICZ 0,1nM**	113,417	2,731	3	0,350237
**FICZ 1nM**	58,174	10,643	3	0,040578
**FICZ 10nM**	9,124	6,480	3	0,002295
**FICZ 100nM**	-1,157	5,525	3	0,001469
**Vem 0,1mM**	93,537	6,750	3	0,649736
**Vem 1μM**	54,926	2,209	3	0,023593
**Vem 10μM**	11,847	6,165	3	0,002534

Values represent % of remaining [3H]TCDD radiactivity to CTR condition

Figure 1c				
	**Median**	**SD**	**n**	**P value**
**CTR (DMSO)**	5	3,158	28	
**TCDD 10nM**	23,5	8,656	18	<0,0001
**Vem 1μM**	4	2,376	21	0,0491
non-parametric two-tailed Mann Whitney test CTR vs TCDD or Vem				

Figure 1d				
	**Mean**	**SD**	**n**	**P value**
**CTR (DMSO)**	1	0	3	
**TCDD 10nM**	3,125	0,38	3	0,000634
**Vem 1μM**	0,458	0,024	3	<0,0001

Values represent Luciferase enrichment relative to CTR condition

Figure 1e				
	**Mean**	**SD**	**n**	**P value**
**CTR (DMSO)**	1	0	6	
**TCDD 10nM**	20,708	3,847	6	<0,0001
**Vem 1μM**	0,693	0,333	6	0,047639

Relative quantitative RNA was normalized to 18S rRNA

Values represent fold change relative to CTR condition

Figure 1f				
	**Mean**	**SD**	**n**	**P value**
**CTR (DMSO)**	1	0	3	
**TCDD 10nM**	100	12	3	0,000139
**Vem 1μM**	-2,2	2,5	3	0,090913

Values % of EROD activity to TCDD induced MCF7 condition

Figure 2

Figure 2h				
Cell lines	IC50	log2 fold change (α signature)	log2 fold change (β signature)	fold change (resistance signature)
501 S	4,679E-08	1	1	1
MM001	9,143E-08	1,059203	0,9751523	2,219208
MM074 S	0,0000001	1,712855	1,252197	2,377848
Mel624	4,679E-07	1,560535	1,07601	1,67031
501 R	5,579E-07	1,611878	0,669492	3,568108
Skmel28 S	5,697E-07	1,986582	0,4181132	2,678657
MM074 R	0,000001	2,625797	0,8070144	3,875775
Skmel28 R	1,75E-06	2,227963	0,0017973	5,052008
Mel165	1,795E-06	2,985939	0,00043556	6,353109
n=1				

Figure 5

Figure 5c			
fold enrichment vs DMSO	Mean TCDD 10nM 7J	Mean TCDD 10nM 14J	n
AhR	0,8608	2,9031	2
GCNT1	0,7411	1,3902	2
BIRC3	0,9165	2,9503	2
THBS1	2,4238	6,9312	2
AXL	7,9785	7,5228	2
ZEB1	0,8286	12,2491	2
LPAR1	1,1260	4,0799	2
TIPARP	1,4299	0,3483	2
NRP1	0,7759	1,0277	2
CYP1A1	5,8179	5,4463	2
NGFR	0,4516	5,1084	2
EGFR	2,3586	2,0499	2

Figure 5e 501Mel S					
	CR-CTR		*CR-AhR*		
Gene	CTR	Vem 1μM	CTR	Vem 1μM	n
AhR	1	0,828	0,311	0,529	2
BCAR3	1	1,490	4,073	2,973	2
AXL	1	0,276	1,120	0,351	2
EGFR	1	0,797	0,062	0,053	2
NRP1	1	0,783	0,949	0,464	2
TEAD4	1	0,552	0,649	0,357	2
LPAR1	1	0,554	0,005	0,015	2
GCNT1	1	0,272	0,292	0,057	2
NGFR	1	0,949	1,105	0,513	2
WDR24	1	1,185	0,627	0,745	2
RPS16	1	0,840	0,004	0,004	2
SNX13	1	1,493	1,136	1,207	2
BIRC3	1	1,138	5,512	3,103	2
ATP10A	1	1,699	0,002	0,001	2
PHB	1	1,276	0,348	0,407	2
OCA2	1	2,727	0,057	0,173	2
Mitf	1	2,054	0,357	0,806	2
TYRP1	1	3,612	0,173	0,192	2
SLC45A2	1	1,788	0,336	0,111	2
GPR143	1	1,719	0,036	0,060	2
Rab27a	1	2,226	1,428	2,313	2
MLPH	1	1,959	1,017	1,416	2
CYP1A1	1	0,192	0,796	0,337	2
AhRR	1	1,713	3,036	5,569	2
PMAIP1	1	0,324	0,294	0,172	2
Relative quantitative RNA was normalized to 18S rRNA					
Values represent fold change relative to CTR condition					

Figure 5f					
	501Mel R		SKMel28 R		
Gene	siAhR	siARNT	siAhR	siARNT	n
AhR	0,175	0,739	0,191	0,413	2
ARNT	0,577	0,297	0,630	0,369	2
ZEB1	0,502	0,125	0,673	0,713	2
GCNT1	0,454	0,556	0,929	0,994	2
BRI3	1,086	1,224	0,739	0,790	2
BIRC3	0,178	0,539	0,656	0,469	2
PHB	0,854	0,654	0,941	0,744	2
SNX13	0,568	0,553	0,727	0,663	2
ATP10A	0,501	0,637	1,981	5,141	2
PCDH7	0,998	1,309	1,263	1,333	2
TEAD4	6,589	9,673	1,030	1,297	2
LPAR1	0,550	0,544	1,831	1,473	2
HMGXB3	0,532	0,590	0,675	0,787	2
EGFR			0,660	0,796	2
NGFR	1,361	0,652	2,806	2,535	2
AXL	0,438	1,045	0,605	0,656	2
NRP1	5,065	1,157	1,418	1,261	2
AhRR	0,867	0,985	0,781	1,325	2

Relative quantitative RNA was normalized to 18S rRNA

Values represent fold change relative to siCTR condition (siNT1)

Figure 6

Figure 6a								
fold enrichment vs DMSO	OCA2	SD	n	P value vs CTR (DMSO)	CYP1A1	SD	n	P value vs CTR (DMSO)
Vem	4,194	0,356	3	9,961E-05	0,693	0,333	3	0,18579
PLX7904	4,079	0,842	3	3,174E-03	0,387	0,087	3	0,00026
PLX8394	4,483	0,145	3	1,990E-06	0,685	0,377	3	0,22180
Dab	3,909	0,391	3	2,080E-04	0,771	0,419	3	0,39668
TCDD	1,633	0,081	3	1,696E-04	20,708	3,847	3	0,00089
BaP	1,579	0,109	3	7,636E-04	13,392	5,022	3	0,01291
Lkyn	0,861	0,165	3	2,178E-01	0,982	0,251	3	0,90657
FICZ	1,846	0,046	3	5,891E-06	5,276	0,507	3	0,00013
CH-223191	0,226	0,054	3	1,550E-05	0,402	0,291	3	0,02362
RSV	0,960	0,057	3	2,879E-01	0,910	0,128	3	0,28786

Relative quantitative RNA was normalized to 18S rRNA

Figure 6c					
CYP1A1	Mean	SD	n	P value vs CTR	P value vs TCDD
CTR (DMSO)	0,873	0,180	3		
TCDD 10nM	9,139	1,077	3	0,0001951	
RSV 5mM	1,763	0,008	3	0,0010143	
RSV 5mM + TCDD 10nM	1,209	0,256	3	0,1365030	0,0002422

Relative quantitative RNA was normalized to 18S rRNA

Values represent fold change relative to CTR condition

Figure 6f					
OCA2	Mean	SD	n	P value vs CTR	P value vs TCDD
CTR (DMSO)	0,960	0,057	3		
Vem 1μM	4,194	0,356	3	9,967048E-05	
RSV 5mM	0,654	0,207	3	6,898830E-02	
RSV 5mM + Vem 1mM	1,040	0,061	3	1,717660E-01	1,109420E-04

Relative quantitative RNA was normalized to 18S rRNA

Values represent fold change relative to CTR condition

Figure 6h				
	Mean	SD	n	
**0h**	0,357	0,023	4	**CTR (DMSO)**
**24h**	0,739	0,034	4	
**48h**	1,557	0,049	4	
**72h**	1,886	0,066	4	
**0h**	0,334	0,019	4	**Vem 1μM**
**24h**	0,540	0,005	4	
**48h**	0,748	0,030	4	
**72h**	0,785	0,048	4	
**0h**	0,372	0,028	4	**RSV 5mM**
**24h**	0,624	0,027	4	
**48h**	1,131	0,019	4	
**72h**	1,228	0,059	4	
**0h**	0,268	0,029	4	**RSV 5mM + Vem 1mM**
**24h**	0,554	0,035	4	
**48h**	0,807	0,091	4	
**72h**	0,799	0,043	4	

Figure 6i					
**mean % of viability of SK28 S cells**	**1**	**2**	**3**	**4**	
**0,0**	106,8	113,2	99,2	106,9	**CTR**
**1,0E-07**	88,8	96,1	105,8	117,6	
**5,0E-07**		63,5	81,4	82,2	
**1,0E-06**		49,6	59,3	58,2	
**5,0E-05**	34,9	42,5	57,8	56,2	
**1,0E-05**	18,1	27,5	28,6	35,5	
**0,0**	92,4	98,8	105,8	103,0	**RSV 7days**
**1,0E-07**		53,1	62,6	63,7	
**5,0E-07**	24,3	30,6	40,5	41,6	
**1,0E-06**	19,4	25,3	32,3	36,2	
**5,0E-05**	12,6	15,5	19,8	20,4	
**1,0E-05**	10,6	10,8	12,9	12,4	

Figure 6j		
**IC50 (M)**	**CTR**	**RSV 7days**
**CTR (DMSO)**	4,68E-08	6,58E-08
**Vem 1μM**	5,58E-07	8,20E-08
**RSV 5mM**	5,70E-07	1,16E-07
**RSV 5mM + Vem 1mM**	1,75E-06	1,54E-06

IC50 values have been calculated using PRISM Graph Pad

Figure 6k						
**% of persistent cells**	**1**	**2**	**3**	**4**	**P value vs CTR**	
**501Mel S**	28,6	30,2	28,8	28,0		**CTR**
**501Mel R**	38,3	36,6	35,2	43,5		
**SKMel28 S**	34,9	42,5	57,8	56,2		
**SKMel28 R**	17,4	22,2	22,7	25,1		
**501Mel S**	14,7	15,3	15,4	15,4	1,406575E-07	**RSV 7days**
**501Mel R**	21,2	23,5	25,6	27,0	6,920340E-04	
**SKMel28 S**	12,6	15,5	19,8	20,4	1,857980E-03	
**SKMel28 R**	24,1	21,3	27,1	21,7	4,584530E-01	

% of persistent cells have been obtained from viability curves

Figure 6l								
	Dabrafenib (30mg/Kg daily)							
**Days after treatment/ tumor volume (mm3)**	#1	#2	#3	#4	#5	#6	#7	#8
14	649,757	748,9422	1176	949,5294	482,5809	389,1881	888	342,974

Figure 6m								
	Dabrafenib (30mg/Kg daily)							
**Max tumor volume/ days to reach**	#1	#2	#3	#4	#5	#6	#7	#8
800 mm3	16	16	14	14	20	20	14	16

Figure 6l							
	Dabrafenib (30mg/Kg daily) + RSV (40mg/kg daily)						
Days after treatment/ tumor volume (mm3)	#1	#2	#3	#4	#5	#6	#7
14	427,9034	462,071	307,4376	294,9526	384,9775	225,8256	352,0565

Figure 6m							
	Dabrafenib (30mg/Kg daily) + RSV (40mg/kg daily)						
Max tumor volume/ days to reach	#1	#2	#3	#4	#5	#6	#7
800 mm3	20	20	24	22	26	30	

Supplementary Figure 1

Supplementary Figure 1a				
%	Mean	SD	n	P value
**CTR**	100	21,821	3	1
**FICZ 10nM**	9,124	6,480	3	0,002295
**Dab 1μM**	82,590	8,665	3	0,268346
**Dab 10μM**	54,800	14,480	3	0,040358
**Dab 100μM**	8,257	6,845	3	0,002254
**PLX7904 1μM**	109,550	34,563	3	0,706421
**PLX7904 10μM**	63,662	10,763	3	0,060888
**PLX7904 100μM**	34,577	23,865	3	0,024801
**PLX8394 1μM**	80,469	12,622	3	0,250708
**PLX8394 10μM**	88,520	24,097	3	0,573809
**PLX8394 100μM**	20,291	13,543	3	0,005785
Values represent % of remaining [3H]TCDD radiactivity to CTR condition				

Supplementary Figure 1d				
	Mean	SD	n	P value
**CTR (DMSO)**	1		3	
**Dab 100nM**	0,705	0,141	3	0,022082
**PLX7904 1μM**	0,232	0,022	3	<0,0001
**PLX8394 1μM**	0,192	0,017	3	<0,0001
Values represent Luciferase enrichment relative to CTR condition				

Supplementary Figure 1e				
	Mean	SD	n	P value
**CTR (DMSO)**	1		6	
**Dab 100nM**	0,771	0,419	6	0,209559
**PLX7904 1μM**	0,387	0,087	6	<0,0001
**PLX8394 1μM**	0,685	0,377	6	<0,0001
Relative quantitative RNA was normalized to 18S rRNA				
Values represent fold change relative to CTR condition				

Supplementary Figure 1f				
	Mean	SD	n	P value
**CTR (DMSO)**	1	0	3	
**Dab 100nM**	0,3	0,5	3	0,158302
**PLX7904 1μM**	2,1	2,6	3	0,504314
**PLX8394 1μM**	-1,8	2,8	3	0,158302
Values % of EROD activity to TCDD induced MCF7 condition				

Supplementary Figure 2

Supplementary Figure 2b					
	Mean	SD	n	P value	
**0**	1	0	3		**Vem 1μM**
**6**	1,226	0,060	3	0,053706	
**12**	2,666	0,356	3	0,001621	
**24**	3,249	0,149	3	<0,0001	
**48**	2,197	0,236	3	0,00143	
**0**	1	0	3		**Dab 100nM**
**6**	1,416	0,097	3	0,167731	
**12**	3,430	0,060	3	<0,0001	
**24**	4,545	0,074	3	<0,0001	
**48**	6,275	0,476	3	<0,0001	
Relative quantitative RNA was normalized to 18S rRNA					
Values repRSVent fold change relative to CTR condition					

Supplementary Figure 2c					
%	Mean	SD	n*	P value	
**CTR DMSO**	100	29,772	2		***AhR***
**sh*AhR*-1 DMSO**	44,78	7,09	3	0,04457	
**sh*AhR*-2 DMSO**	17,18	0,84	4	0,003032	
**CTR Vem 1μM**	100	6,71	2		***OCA2***
**shAhR-1 Vem 1μM**	69,69	13,05	3	0,104152	
**shAhR-2 Vem 1μM**	40,69	7,76	4	0,010056	
**CTR TCDD 10nM**	100	30,06	2		***CYP1A1***
**shAhR-1 TCDD 10nM**	52,82	7,76	3	0,067992	
**shAhR-2 TCDD 10nM**	39,47	5,73	4	0,011546	
n* number of cellular clones by shRNA					
Relative quantitative RNA was normalized to 18S rRNA					
Values represent % of enrichment to CTR condition					

Supplementary Figure 2d				
	Mean	SD	n	P value
**IgG**	1	0	3	
**Ac anti-AhR**	2,01	0,059	3	0,0011
Values represent relative occupancy onto OCA2 promoter to CTR condition				

Supplementary Figure 2e				
	Mean	SD	n	P value
**CTR**	1	0	3	
**Vem 1μM**	2,89	0,17	3	<0,0001
**TCDD 10nM**	1,171	0,19	3	0,212
**CH-223191 10μM**	0,833	0,14	3	
**CH-223191 10μM + Vem 1μM**	0,724	0,141	3	0,254
**CH-223191 10μM + TCDD 10nM**	0,68	0,061	3	0,013
**Dab 100nM**	3,032	0,263	3	0,000181
**PLX7904 1μM**	4,422	0,020	3	<0,0001
**PLX8394 1μM**	6,323	0,874	3	0,000456
**CH-223191 10μM + Dab 100nM**	0,623	0,017	3	<0,0001
**CH-223191 10μM + PLX7904 1μM**	0,804	0,159	3	0,769766
**CH-223191 10μM + PLX8394 1μM**	0,995	0,068	3	0,015179
Values represent Luciferase enrichment relative to CTR condition				

Supplementary Figure 2f				
	Mean	SD	n	P value
CTR	0,477	0,087	3	
Vem 1µM	0,339	0,039	3	0,066821
TCDD 10nM	0,69	0,191	3	0,156043

Values represent Luciferase enrichment relative to CTR condition

Supplementary Figure 2g					
	Mean	SD	n	P value	
CTR DMSO	0,961	0,055	3		*OCA2*
CTR Vem 1µM	3,626	0,166	3	<0,0001	
siARNT DMSO	0,705	0,020	3	0,001555	
siARNT Vem 1µM	3,515	0,358	3	0,000258	
CTR TCDD 10nM	1	0,073	3	0,181298	
siARNT TCDD 10nM	0,488	0,088	3	0,001376	
CTR DMSO	0,901	0,140	3		*CYP1A1*
CTR Vem 1µM	0,237	0,010	3	0,001197	
siARNT DMSO	0,553	0,092	3	0,022754	
siARNT Vem 1µM	0,238	0,016	3	0,001219	
CTR TCDD 10nM	20,68	2,518	3	0,00017	
siARNT TCDD 10nM	3,19	1,433	3	0,051182	

Relative quantitative RNA was normalized to 18S rRNA

Values represent fold change relative to CTR condition

Supplementary Figure 4

	Supplementary Figure 4 OCA2 expression				
		Mean	SD	n	P value
Fig 4a	CTR (DMSO)	1	0	6	
	Vem 1µM	4,194	0,356	6	<0,0001
	PLX7904 1µM	4,079	0,842	6	<0,0001
	PLX8394 1µM	4,483	0,145	6	<0,0001
	Dab 100nM	3,909	0,391	6	<0,0001
	TCDD 10nM	1,633	0,081	6	<0,0001
Fig 4b	Tram 1µM (co-treatment)	Mean	SD	n	P value
	CTR (DMSO)	3,882	0,078	3	<0,0001
	Vem 1µM	3,470	0,052	3	<0,0001
	PLX7904 1µM	4,009	0,123	3	<0,0001
	PLX8394 1µM	4,128	0,569	3	<0,0001
	Dab 100nM	4,028	0,350	3	0,00012
Fig 4c	Cob 1µM (co-treatment)	Mean	SD	n	P value
	CTR (DMSO)	4,025	0,113	3	<0,0001
	Vem 1µM	3,862	0,060	3	<0,0001
	PLX7904 1µM	3,658	0,190	3	<0,0001
	PLX8394 1µM	3,251	0,271	3	<0,0001
	Dab 100nM	3,402	0,036	3	<0,0001
Fig 4d	CH-223191 10µM (2h before)	Mean	SD	n	P value
	CTR (DMSO)	0,226	0,054	3	<0,0001
	Vem 1µM	0,322	0,008	3	<0,0001
	PLX7904 1µM	0,305	0,053	3	<0,0001
	PLX8394 1µM	0,765	0,103	3	<0,0001
	Dab 100nM	0,381	0,052	3	<0,0001
	TCDD 10nM	0,194	0,023	3	<0,0001
Fig 4e	TCDD 10nM (2h before)	Mean	SD	n	P value
	CTR (DMSO)	1	0	3	
	Vem 1µM	1,317	0,243	3	0,010665
	PLX7904 1µM	0,965	0,015	3	<0,0001
	PLX8394 1µM	0,849	0,119	3	0,012332
	Dab 100nM	1,102	0,171	3	0,160549
	TCDD 10nM	1,633	0,081	6	<0,0001

	Supplementary Figure 4 CYP1A1 expression				
		Mean	SD	n	P value
Fig 4a	DMSO	1	0	6	
	Vem 1μM	0,693	0,333	6	0,047639
	PLX7904 1μM	0,387	0,087	6	<0,0001
	PLX8394 1μM	0,685	0,377	6	0,068138
	Dab 100nM	0,771	0,419	6	0,209559
	TCDD 10nM	20,708	3,847	6	<0,0001
Fig 4a	Tram 1μM (co-treatment)	Mean	SD	n	P value
	DMSO	0,572	0,033	3	<0,0001
	Vem 1μM	0,418	0,092	3	0,000390
	PLX7904 1μM	1,802	0,217	3	0,003044
	PLX8394 1μM	1,636	0,051	3	<0,0001
	Dab 100nM	1,922	0,059	3	<0,0001
Fig 4a	Cob 1μM (co-treatment)	Mean	SD	n	P value
	DMSO	0,547	0,000	3	<0,0001
	Vem 1mM	0,688	0,203	3	0,056414
	PLX7904 1mM	0,605	0,133	3	0,006688
	PLX8394 1mM	0,614	0,025	3	<0,0001
	Dab 100nM	0,659	0,036	3	<0,0001
Fig 4a	CH-223191 10μM (2h before)	Mean	SD	n	P value
	DMSO	0,402	0,291	3	<0,0001
	Vem 1μM	0,216	0,128	3	<0,0001
	PLX7904 1μM	0,452	0,165	3	<0,0001
	PLX8394 1μM	0,415	0,003	3	<0,0001
	Dab 100nM	0,552	0,054	3	<0,0001
	TCDD 10nM	0,556	0,040	3	<0,0001
Fig 4a	TCDD 10nM (2h before)	Mean	SD	n	P value
	DMSO	1	0	3	
	Vem 1mM	0,917	0,482	3	0,663347
	PLX7904 1mM	1,740	0,527	3	0,007485
	PLX8394 1mM	0,475	0,156	3	<0,0001
	Dab 100nM	0,864	0,003	3	<0,0001
	TCDD 10nM	20,708	3,847	6	<0,0001

Supplementary Figure 4f Competition assay using XRE luc after activation by TCDD (5nM)							
% XRE Luc activity compared to control (TCDD 5nM alone)							
Antagonist concentration (M)	Vemurafenib				CH-223191		
0,00E+00	87,4451	100	100,0296	100	100	85,99702	93,45161
5,00E-08	94,48193	72,20885	75,83105	82,40413	71,51735	70,93938	77,71037
1,00E-07	61,27043	96,75644	97,21919	65,9942	72,15801	76,21183	61,91182
5,00E-07	54,39727	67,59219	71,03971	55,21734	49,78279	49,9878	54,46649
1,00E-06	32,22122	62,25197	58,39666	40,73503	37,57643	40,193	35,33511
2,00E-06	23,34847	62,62482	58,2317	38,994	38,0734	32,97725	39,82437
5,00E-06	13,19083	22,54103	27,29546	11,5572	16,66262	20,88602	18,82049
1,00E-05		17,6874	17,58166		13,87465	17,51776	19,2419
2,00E-05	4,012937	6,330139	8,372258	2,850804	13,69989	16,12879	18,89792

Supplementary Figure 4f XRE binding assay (PLS unit)								
	Competition with DMSO				Competition with Vem (1 μM)			
	Mean	SD	n	P value	Mean	SD	n	P value
CTR (DMSO)	6899,94	775,1472	3		5545,36	1294,875	3	
TCDD	24952,97	3224,679	3	0,19500	9952,193	1755,89	3	0,00210

Supplementary Figure 6

Supplementary Figure 6b				
	CTR	Vem (1μM) for 6h	TCDD (10nM) for 1h	AhR KO
**TIPARP**	1,04	1,18	9,63	0,53
**NRP1**	4,61	5,77	52,31	0,02
**GCNT1**	1,49	1,51	9,79	0,04
**TEAD4**	7,99	2,57	20,12	0,00
**EGFR**	0,09	0,09	0,09	0,09
**ZEB1**	0,12	0,12	0,12	0,12

Values represent relative occupancy of AhR (%) onto different genes promoter compared to Input (10%) (n=1)

Supplementary Figure 8

Supplementary Figure 8a					
OCA2	Mean	SD	n	P value vs CTR	P value vs BRAFi
**CTR (DMSO)**	0,910	0,128	3		
**Dab 100nM**	3,909	0,391	3	0,000205	
**RSV 5mM**	0,654	0,207	3	0,068988	
**RSV 5mM + Dab 100nM**	1,306	0,285	3	0,107584	0,000735
**CTR (DMSO)**	0,910	0,128	3		
**PLX7904 1μM**	5,626	0,028	3	<0,0001	
**RSV 5mM**	0,654	0,207	3	0,068988	
**RSV 5mM + PLX7904 1mM**	2,313	0,793	3	0,041923	0,001938
**CTR (DMSO)**	0,910	0,128	3		
**PLX8394 1μM**	5,397	0,972	3	0,001398	
**RSV 5mM**	0,654	0,207	3	0,068988	
**RSV 5mM + PLX8394 1mM**	2,963	0,720	3	0,0085964	0,025281

Relative quantitative RNA was normalized to 18S rRNA

Values represent fold change relative to CTR condition

Supplementary Figure 8b				
	Mean	SD	n	
**0h**	0,357	0,023	4	**CTR (DMSO)**
**24h**	0,739	0,034	4	
**48h**	1,557	0,049	4	
**72h**	1,886	0,066	4	
**0h**	0,298	0,032	4	**Dab 100nM**
**24h**	0,536	0,034	4	
**48h**	0,697	0,065	4	
**72h**	0,478	0,051	4	
**0h**	0,372	0,028	4	**RSV 5mM**
**24h**	0,624	0,027	4	
**48h**	1,131	0,019	4	
**72h**	1,228	0,059	4	
**0h**	0,295	0,031	4	**RSV 5mM + Dab 100nM**
**24h**	0,560	0,077	4	
**48h**	0,729	0,077	4	
**72h**	0,573	0,102	4	
**0h**	0,313	0,055	4	**PLX7904 1μM**
**24h**	0,555	0,093	4	
**48h**	0,641	0,020	4	
**72h**	0,501	0,086	4	
**0h**	0,337	0,044	4	**RSV 5mM + PLX7904 1mM**
**24h**	0,581	0,037	4	
**48h**	0,704	0,036	4	
**72h**	0,691	0,116	4	
**0h**	0,344	0,010	4	**PLX8394 1μM**
**24h**	0,549	0,006	4	
**48h**	0,657	0,049	4	
**72h**	0,566	0,097	4	
**0h**	0,362	0,013	4	**RSV 5mM + PLX8394 1mM**
**24h**	0,552	0,044	4	
**48h**	0,670	0,048	4	
**72h**	0,541	0,109	4	

Supplementary Figure 8c								
	Dabrafenib (30mg/Kg daily)							
Days after treatment/ tumor volume (mm3)	#1	#2	#3	#4	#5	#6	#7	#8
0	228,5784	145,9038	163,8505	453,9627	155,5811	192,3251	181,9278	212,3261
2	400,5609	236,715	273,5388	785,0981	256,5916	253,1611	358,7481	145,4938
4	445,3407	213,7754	309,2954	699,532	177,139	273,0444	362,9818	193,5539
6	446,8115	161,346	443,423	463,8883	246,3018	383,0661	237,6396	177,0281
8	513,4908	171,8426	284,6789	431,2435	181,2704	267,6297	435,4947	232,2182
10	586,1133	278,1571	500,9821	579,2245	207,8737	210,371	490,1361	281,6341
12	563,4523	376,8195	646,1647	673,1277	338,8754	404,3548	681,4609	258,4264
14	649,7568	748,9422	1175,886	949,5294	482,5809	389,1881	888,3819	342,974
16	847,4342	779,0064		1553,923	447,7659	629,5346		847,3161
18	761,9802	1159,95			616,3477	504,2755		943,5343

Supplementary Figure 8c							
	Dabrafenib (30mg/Kg daily) + RSV (40mg/kg daily)						
Days after treatment/ tumor volume (mm3)	#1	#2	#3	#4	#5	#6	#7
0	221,4501	194,1715	275,3976	224,8464	398,1097	244,3485	244,1665
2	257,4719	189,4749	168,3477	286,4874	250,3039	174,2905	275,6007
4	237,0102	232,694	177,4051	218,9116	248,9659	154,4211	240,943
6	188,7079	186,8194	198,6457	332,4404	215,6226	134,3872	226,298
8	321,1533	219,9162	297,2921	210,2907	221,5042	377,3848	253,0369
10	375,9003	388,4406	301,4202	433,4024	340,393	274,9919	243,5691
12	274,3714	399,4749	321,6159	557,6225	483,5854	289,065	345,9168
14	427,9034	462,071	307,4376	294,9526	384,9775	225,8256	352,0565
16	609,4879	597,6372	281,6883	565,3165	435,1711	359,6186	247,3423
18	545,4904	719,8815	544,3751	517,2294	471,3379	456,4025	380,7241
